# Supplementary material for: Response of in situ root phenotypes to potassium stress in cotton
Source: PeerJ. 2023 Jun 21;11:e15587. doi: 10.7717/peerj.15587 (PMC10290453; doi:10.7717/peerj.15587)
Supplement: Table S1 [file peerj-11-15587-s001.docx]

**Supplementary Table**

**TABLE S1.** **Differences in cotton yield in different cotton organs.**

| Treatment | g/Plant | | |
| --- | --- | --- | --- |
|  | Seed | Lint | Unginned cotton |
| LK | 2.51 ± 0.38 c | 2.19 ± 0.27 b | 4.70 ± 0.48 c |
| MK | 4.13 ± 0.52 a | 3.35 ± 0.38 a | 7.48 ± 0.41 a |
| HK | 3.63 ± 0.57 b | 3.21 ± 0.41 a | 6.83 ± 0.55 b |

Note: Statistical significant differences (*p < 0.05*) are shown as different letters.
