# Supplementary material for: Response of in situ root phenotypes to potassium stress in cotton
Source: PeerJ. 2023 Jun 21;11:e15587. doi: 10.7717/peerj.15587 (PMC10290453; doi:10.7717/peerj.15587)
Supplement: Table S2 [file peerj-11-15587-s002.docx]

**Supplementary Table**

**TABLE S2.** **Differences in potassium accumulation in different cotton organs.**

| Treatment | K accumulation rates (mg/plant) | | | | Total K (mg/plant) |
| --- | --- | --- | --- | --- | --- |
|  | Root | Stem | Leaf | Boll |  |
| LK | 9.66±0.71 b | 16.75±2.14 c | 13.51±0.76 c | 144.22±10.45 b | 184.13±9.60 c |
| MK | 15.82±1.52 a | 26.76±2.39 b | 21.85±2.13 b | 206.46±13.63 a | 270.89±12.64 b |
| HK | 14.75±1.06 a | 44.59±4.42 a | 52.35±4.39 a | 199.08±15.58 a | 310.81±15.59 a |

Note: Statistical significant differences (*p < 0.05*) are shown as different letters.
