# Supplementary figures and images for: Response of in situ root phenotypes to potassium stress in cotton
Source: PeerJ. 2023 Jun 21;11:e15587. doi: 10.7717/peerj.15587 (PMC10290453; doi:10.7717/peerj.15587)

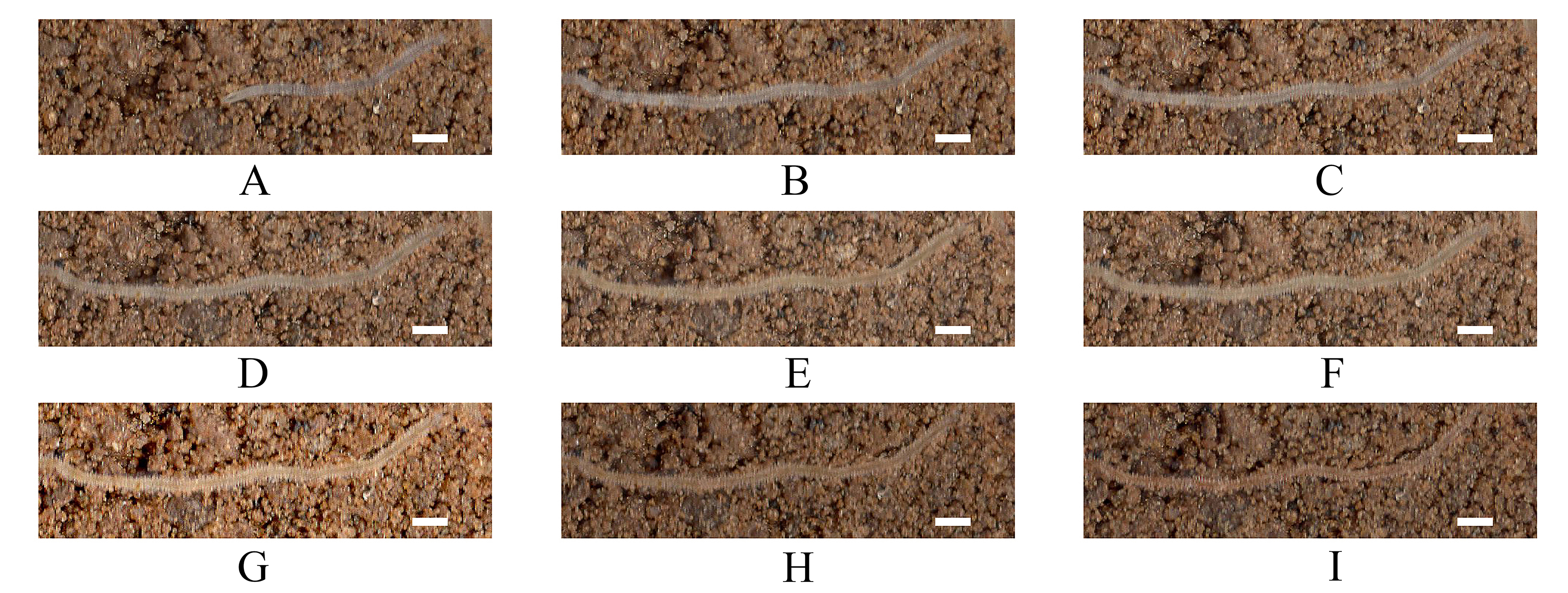

Supplement: Figure S1 — (A–I) represent the 1st, 3rd, 5th, 10th, 20th, 30th, 40th, 50th, and 60th day root images of lateral root appearance, respectively. Scale bar, 1 mm. [file peerj-11-15587-s003.jpg]

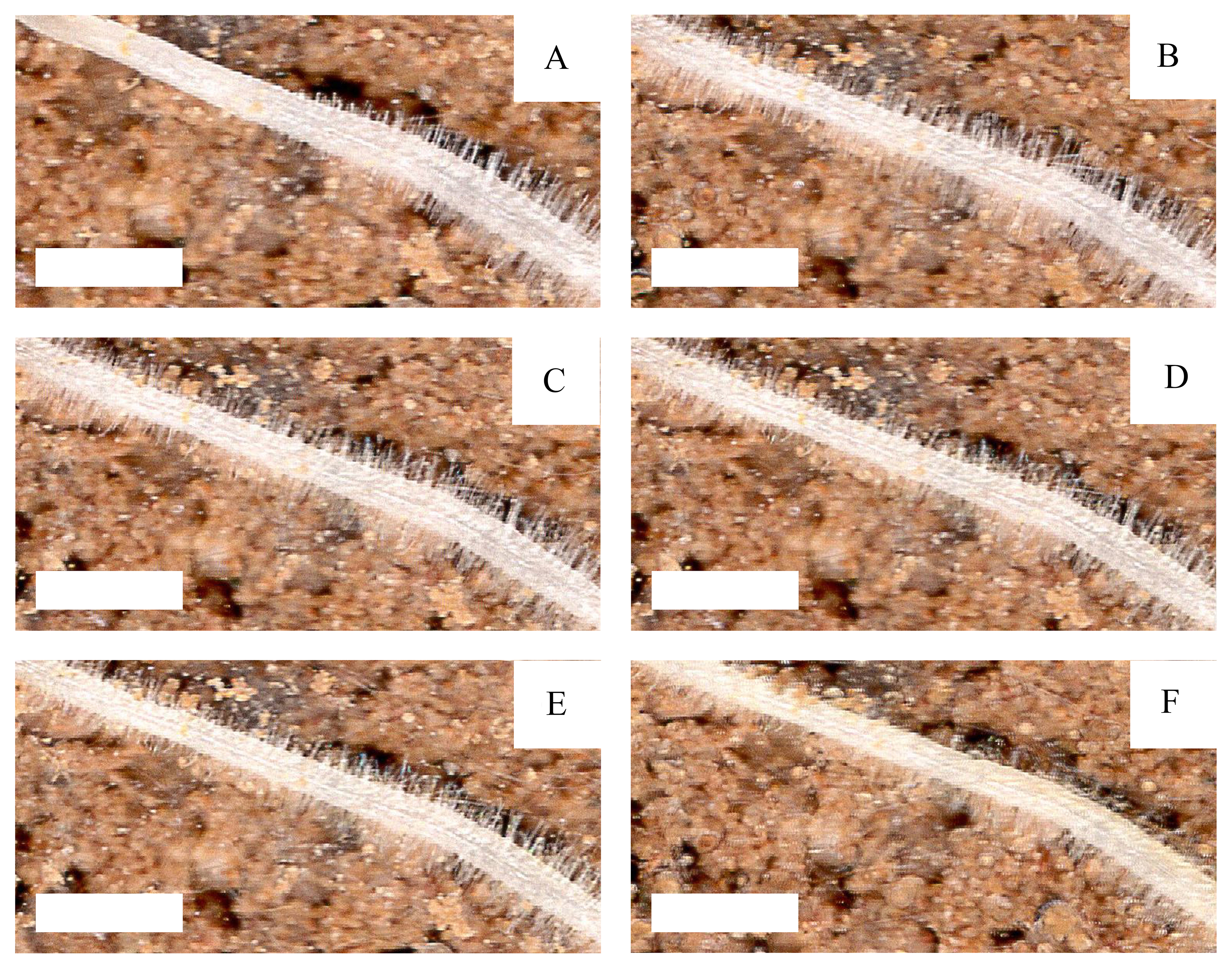

Supplement: Figure S2 — (A–F) represent the images of the roots on days 1, 7, 13, 19, 25, and 31 of lateral root appearance, respectively. Scale bar, 1 mm. [file peerj-11-15587-s004.jpg]
